# Histone chaperone ASF1 acts with RIF1 to promote DNA end joining in BRCA1-deficient cells

**DOI:** 10.1016/j.jbc.2022.101979

**Published:** 2022-04-25

**Authors:** Mengfan Tang, Zhen Chen, Chao Wang, Xu Feng, Namsoo Lee, Min Huang, Huimin Zhang, Siting Li, Yun Xiong, Junjie Chen

**Affiliations:** Department of Experimental Radiation Oncology, Unit 1052, The University of Texas MD Anderson Cancer Center, Houston, Texas, USA

**Keywords:** ASF1, RIF1, BRCA1, 53BP1, PARPi, homologous recombination, ATM, mutated in ataxia–telangiectasia [A–T], DMEM, Dulbecco’s modified Eagle's medium, DSB, double-strand break, FL, full length, HR, homologous recombination, IR, ionizing radiation, MDC1, mediator of DNA damage checkpoint protein 1, MRN, MRE11–RAD50–NBS1, MS, mass spectrometry, NETN, NaCl, EDTA, Tris–HCl, NP-40, NHEJ, nonhomologous end joining, PARP, poly (ADP-ribose) polymerase, PARPi, PARP inhibitor, PTIP, pax transactivation domain–interacting protein, RIF1, replication timing regulatory factor 1, RPA, replication protein A, SFB, S protein, FLAG, and streptavidin-binding peptide, sgRNA, single-guide RNA, 293T, human embryonic kidney 293T cell line, TAP, tandem affinity purification, TLK, Tousled-like kinase, TRF2, telomeric repeat–binding protein 2

## Abstract

Replication timing regulatory factor 1 (RIF1) acts downstream of p53-binding protein 53BP1 to inhibit the resection of DNA broken ends, which plays critical roles in determining the DNA double-strand break repair pathway choice between nonhomologous end joining and homologous recombination (HR). However, the mechanism by which this choice is made is not yet clear. In this study, we identified that histone chaperone protein ASF1 associates with RIF1 and regulates RIF1-dependent functions in the DNA damage response. Similar to loss of RIF1, we found that loss of ASF1 resulted in resistance to poly (ADP-ribose) polymerase (PARP) inhibition in BRCA1-deficient cells with restored HR and decreased telomere fusion in telomeric repeat–binding protein 2 (TRF2)-depleted cells. Moreover, we showed that these functions of ASF1 are dependent on its interaction with RIF1 but not on its histone chaperone activity. Thus, our study supports a new role for ASF1 in dictating double-strand break repair choice. Considering that the status of 53BP1–RIF1 axis is important in determining the outcome of PARP inhibitor–based therapy in BRCA1- or HR-deficient cancers, the identification of ASF1 function in this critical pathway uncovers an interesting connection between these S-phase events, which may reveal new strategies to overcome PARP inhibitor resistance.

Double-strand breaks (DSBs) are highly deleterious lesions that, if unrepaired or misrepaired, can lead to genomic instability, tumorigenesis, and cell death (1, 2, 3). To preserve genome integrity, cells repair DSBs *via* two evolutionary conserved mechanisms: nonhomologous end joining (NHEJ) and homologous recombination (HR) (4, 5, 6). NHEJ can occur throughout cell-cycle phases *via* the direct ligation of two blunt DNA ends with simple end trimming (4, 7). DSB repair by HR is restricted in the S and G2 phases because it needs unbroken sister chromatids as a repair template (8, 9). HR is initiated *via* two sequential steps of 5′-to-3′ DNA end resection by MRN (MRE11–RAD50–NBS1)–CtIP, EXO1–DNA2, and other nucleases to generate 3′ ssDNA overhang (5, 10, 11, 12, 13). The 3′ ssDNA overhang is rapidly covered by replication protein A (RPA), which is then replaced by recombinase RAD51 with the help of the BRCA1–PALB2–BRCA2 recombination mediator complex (14, 15, 16, 17, 18, 19, 20, 21, 22). The resulting RAD51 nucleoprotein filaments conduct homology search and strand invasion followed by DNA synthesis at the resected strand to precisely repair the broken DNA (10, 15, 22, 23).

Whether a given DSB will be processed by NHEJ or HR is determined by DNA end resection. The equilibrium between NHEJ and HR is mainly established by 53BP1 and BRCA1 (24, 25, 26). Upon DSB induction, 53BP1 rapidly accumulates at DSBs by recognizing two histone modifications: H4K20 dimethylation and H2AK15 monoubiquitination (27, 28). Then ATM (mutated in ataxia–telangiectasia [A–T])-dependent phosphorylation of 53BP1 promotes the recruitment of PTIP (pax transactivation domain–interacting protein) and RIF1 to facilitate NHEJ (29, 30, 31, 32, 33, 34). PTIP recruits Artemis to trim DNA ends and support NHEJ (35), whereas RIF1 recruits the Shieldin complex (SHLD1–SHLD2–SHLD3–REV7) and the CST/polα/primase complex to antagonize BRCA1-dependent end resection, thus promoting NHEJ (26, 36, 37, 38, 39, 40, 41). Conversely, BRCA1 activates DNA end resection and impairs NHEJ by impeding RIF1 retention at DSBs in S-phase cells (29, 30, 32).

RIF1 was originally identified as a telomere-binding protein that negatively regulates telomere length in budding yeast *Saccharomyces cerevisiae* (42, 43, 44). Later, this function was found to be conserved in fission yeast *Schizosaccharomyces pombe* (45). However, in mammalian cells, RIF1 only binds to aberrant telomeres in an ATM–53BP1–dependent manner when telomeres are unprotected and recognized as sites of DNA damage (46, 47). Recent studies showed that in both yeasts and metazoans, RIF1 functions in both the timing of DNA replication and DNA repair (46, 48, 49, 50, 51, 52). RIF1 is a large protein that contains two conserved domains: N-terminal α-helical HEAT repeats, which are required for its localization to sites of DNA damage (29) and two RVxF/SILK motifs at its C terminus, which are implicated in the recruitment of PP1 phosphatase to control the initiation of DNA replication (50). While RIF1 has also been shown to interact with BLM or CSB *via* its C-terminal region, which could regulate DNA replication or DSB repair choice (53, 54, 55), it remains unclear how RIF1 coordinates with different proteins and regulates diverse cellular functions.

In this study, we used CRISPR–Cas9 technology and HR-based knock-in strategy to insert an SFB (S protein, FLAG, and streptavidin-binding peptide) tag at the N terminus of human RIF1 and performed purification of endogenous RIF1–containing protein complexes. We identified ASF1 as a novel binding partner of RIF1, which participates in DNA repair pathway along with RIF1. We showed that the middle region of RIF1 (967–1350) is required for its binding to ASF1A. The N-terminal chaperone domain of ASF1A, but not the key residue critical for its histone chaperone activity, is required for its interaction with RIF1. Depletion of ASF1 leads to resistance to poly (ADP-ribose) polymerase (PARP) inhibitor (PARPi) treatment in BRCA1-deficient cells *via* restoration of HR. Moreover, depletion of ASF1 also decreases telomere fusion observed in telomeric repeat–binding protein 2 (TRF2)-depleted cells, suggesting its role in promoting NHEJ. Furthermore, we found that the binding of ASF1 to RIF1, but not its histone chaperone activity, is required for its role in regulating PARPi sensitivity in BRCA1-deficient cells. Taken together, our study uncovers a new role of ASF1 in DSB repair choice, which is dependent of its interaction with RIF1.

## Results

### ASF1 is a novel RIF1-interacting protein

**Figure 1:**
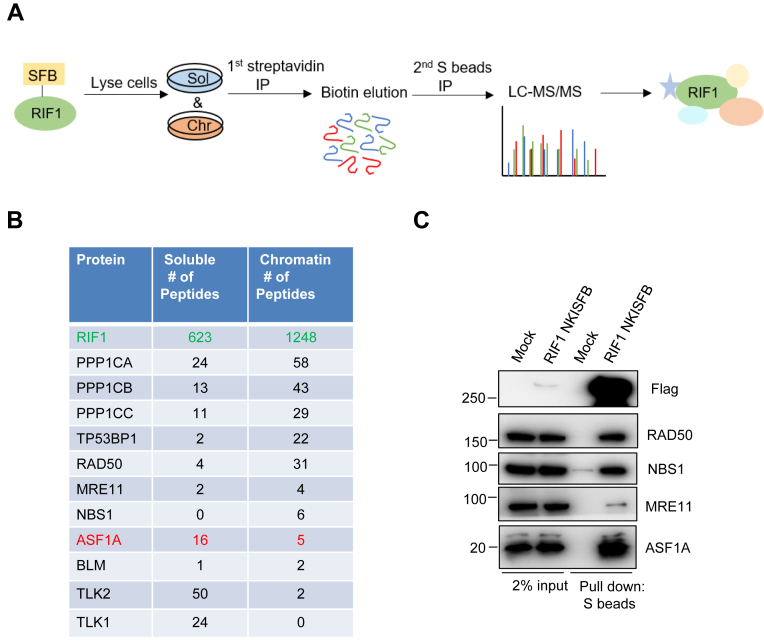
**Identification of RIF1-associated proteins.***A*, strategy for tandem affinity purification (TAP) of 293T RIF1 SFB N-terminal knock-in cells. *B*, selected lists of RIF1-associated proteins in soluble and chromatin fractions analyzed by mass spectrometry. *C*, endogenous RIF1 interacts with ASF1A and MRN complexes. Cell lysates from 293T RIF1 SFB N-terminal knock-in cells were prepared and pulled down with S protein–conjugated beads. Pull-down samples were blotted using antibodies as indicated. MRN, MRE11–RAD50–NBS1; RIF1, replication timing regulatory factor 1; SFB, S protein, FLAG, and streptavidin-binding peptide; 293T, human embryonic kidney 293T cell line.

RIF1 is a large protein that contains 2446 amino acids. We were unable to efficiently uncover RIF1-associated protein complexes because its exogenous expression level *via* any mammalian expression vector we used was quite low. In order to identify RIF1-associated proteins, we utilized an HR-based CRISPR knock-in strategy to insert an SFB tag to the N terminus of RIF1 at its endogenous genomic loci in human embryonic kidney 293T (293T) cells (Fig. S1*A*). Correctly edited clones were identified by genomic PCR (Fig. S1*B*) and Western blotting (Fig. S1*C*) and confirmed by analysis of their localization to DSB foci induced by phleomycin D1 (Zeocin) treatment (Fig. S1*D*). We then carried out tandem affinity purification (TAP) to analyze endogenous RIF1-associated proteins in soluble and chromatin fractions (Fig. 1*A*). As shown in Figure 1*B* and Table S1, we identified several known RIF1-associated proteins such as three isoforms of PP1 phosphatase (PPP1CA, PPP1CB, and PPP1CC) (56, 57) and TP53BP1 (29, 30, 32, 58). Interestingly, these proteins also included several novel RIF1-binding proteins, including ASF1A, Tousled-like kinases (TLK1 and TLK2), and MRN complexes either mainly in soluble or in chromatin fraction. We carried out S protein pull down of SFB-tagged endogenous RIF1 and confirmed the binding of both MRN complexes and ASF1A with RIF1 (Fig. 1*C*). Among those newly identified RIF1-associated proteins, ASF1A showed the strongest binding to RIF1.

ASF1 is an upstream H3–H4 histone chaperone that helps the handover of the H3.1–H4 and H3.3–H4 histones, respectively, to a downstream histone chaperone CAF-1 for replication-dependent chromatin assembly and to another histone chaperone HIRA for replication-independent chromatin assembly (59, 60, 61, 62, 63). Higher eukaryotes contain two paralogs of ASF1, ASF1A, and ASF1B, with significant sequence divergency at their C terminus (64, 65). ASF1 has been reported to participate in diverse regulation of DNA repair. For example, ASF1A promotes H3K56 acetylation by CBP/p300 acetyltransferase, which is required for nucleosome reassembly after DNA repair (66). Codepletion of histone chaperones ASF1A and ASF1B could rapidly induce alternative lengthening of telomeres (ALT) (67). ASF1A promotes NHEJ repair by facilitating phosphorylation of MDC1 (mediator of DNA damage checkpoint protein 1) by ATM at DSBs (68). In response to DSBs, ASF1 also regulates the activation of ATM and DNA-PKcs (69). ASF1 and CAF-1 promote the recruitment of MMS22L/TONSL to ssDNA to load RAD51 during HR in human cells (70). While multiple roles of ASF1 in DNA damage repair have been identified, whether it has a role in RIF1-related function remains to be clarified.

**Figure 2:**
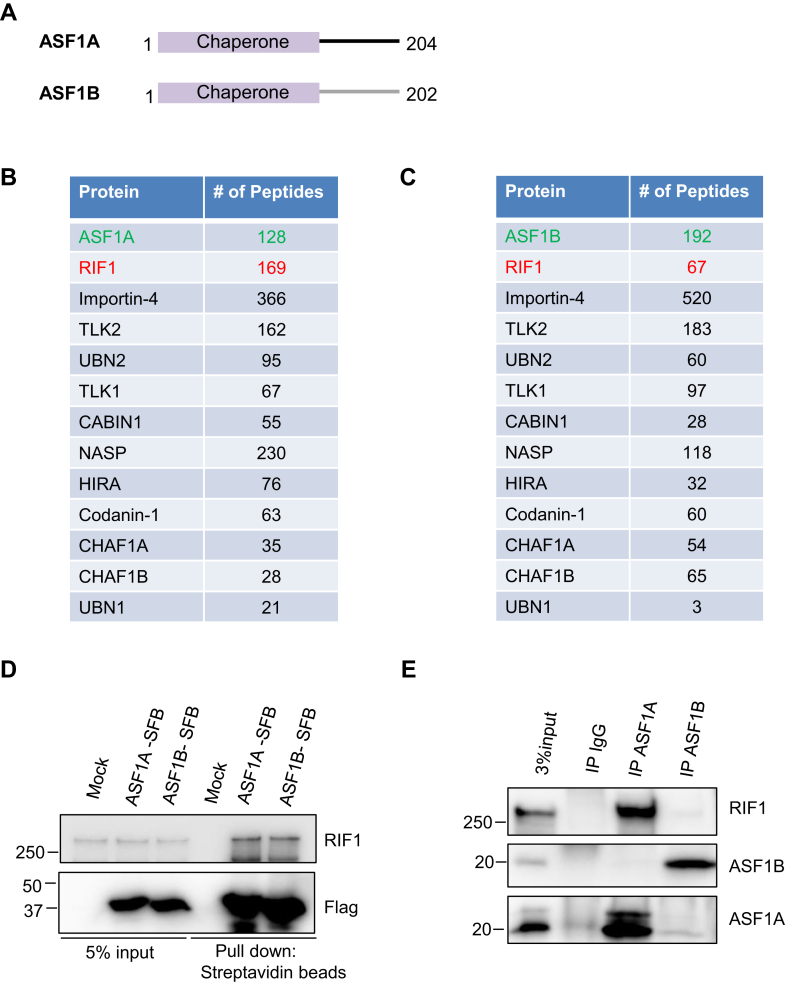
**ASF1A associates with RIF1.***A*, schematic representation of ASF1A and ASF1B protein structure. *B*, selected lists of ASF1A-associated proteins analyzed by mass spectrometry. *C*, selected lists of ASF1B-associated proteins analyzed by mass spectrometry. *D*, mock-transfected 293T cells or 293T cells transfected with constructs encoding SFB-tagged ASF1A or ASF1B were lysed and pulled down with streptavidin-conjugated beads. Pull-down samples were blotted using antibodies as indicated. *E*, cell lysates from 293T cells were immunoprecipitated (IP) with IgG, antibodies recognizing endogenous ASF1A or ASF1B, and the immunoprecipitates were blotted using antibodies as indicated. IgG, immunoglobulin G; RIF1, replication timing regulatory factor 1; SFB, S protein, FLAG, and streptavidin-binding peptide; 293T, human embryonic kidney 293T cell line.

We next performed reverse TAP purification of ASF1A to identify ASF1A-associated proteins. Considering that ASF1B shares similar domain structures with ASF1A (Fig. 2*A*), we also conducted TAP purification of ASF1B. As shown in Figure 2*B* and Table S2, ASF1A TAP purification pulled down many known ASF1A-binding proteins, such as Importin-4, HIRA, CHAF1, TLK1, and TLK2. We also noticed that we identified a lot of RIF1 peptides in our ASF1A purification. Similar to ASF1A purification, ASF1B also pulled down many known ASF1-binding proteins as well as RIF1 (Fig. 2*C* and Table S3). We validated these results by coimmunoprecipitation experiments using overexpressed SFB-tagged ASF1A and ASF1B and showed that both ASF1A and ASF1B could bind to RIF1 (Fig. 2*D*). However, when we performed endogenous coimmunoprecipitation experiments with antibodies recognizing ASF1A or ASF1B, we found that endogenous ASF1A can pull down a proportion of RIF1, whereas the endogenous interaction between ASF1B and RIF1 was barely detectable (Fig. 2*E*). These results indicate that RIF1 binds predominantly to ASF1A at endogenous level. Taken together, we identified ASF1 as a new RIF1-associated protein and suggested a potential role of ASF1—mainly ASF1A—in DNA damage repair through its interaction with RIF1.

### Mapping the interaction domains between RIF1 and ASF1A

**Figure 3:**
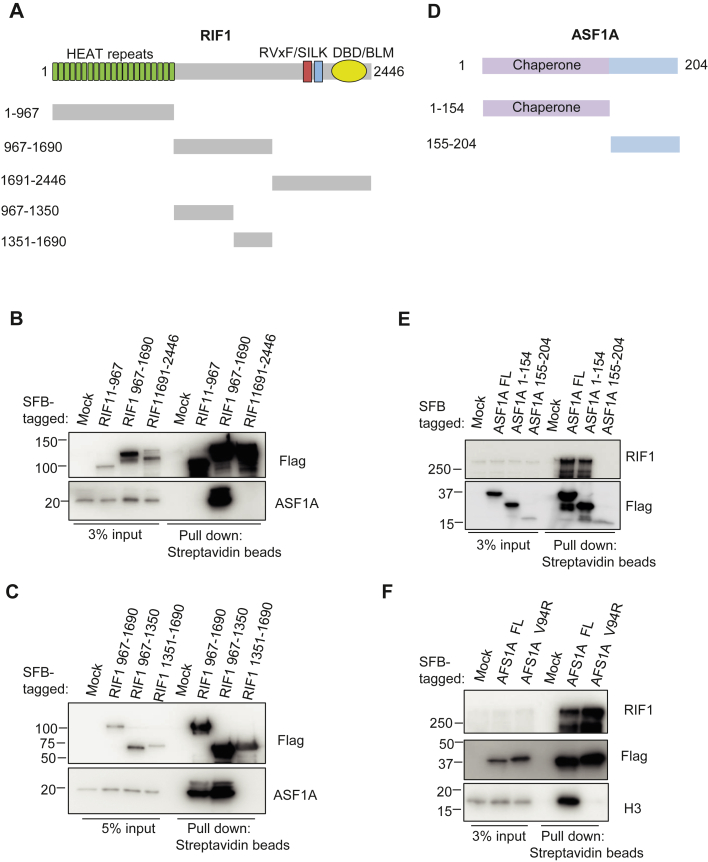
**Mapping the binding regions on RIF1 and ASF1A.***A*, schematic presentation of full-length and truncation mutants of RIF1 used in this study. *B* and *C*, 293T cells were transfected with plasmids encoding various truncation mutants of SFB-tagged RIF1. Pull downs were performed with the use of streptavidin-conjugated beads, and Western blotting was conducted with FLAG and ASF1A antibodies. *D*, schematic presentation of full-length and truncation mutants of ASF1A used in this study. *E*, 293T cells were transfected with plasmids encoding WT or various mutants of SFB-tagged ASF1A. Pull downs were performed with the use of streptavidin-conjugated beads, and Western blotting was conducted with FLAG and RIF1 antibodies. *F*, 293T cells were transfected with plasmids encoding SFB-tagged WT or V94R mutant ASF1A. Pull downs were performed with the use of streptavidin-conjugated beads, and Western blotting was conducted with FLAG and RIF1 antibodies. RIF1, replication timing regulatory factor 1; SFB, S protein, FLAG, and streptavidin-binding peptide; 293T, human embryonic kidney 293T cell line.

Next, we examined which domain of RIF1 is required for its interaction with ASF1A. Because the tagged full length (FL) of RIF1 is difficult to express, we first made three truncation mutants (*i.e.*, residues 1–967, 967–1690, or 1691–2446) of RIF1 and performed coimmunoprecipitation experiments with overexpressed SFB-tagged RIF1 mutants in 293T cells. We found that the region of RIF1 containing residues 967 to 1690 is required for its interaction with ASF1A (Fig. 3, *A* and *B*). We then further divided this region of RIF1, that is, residues 967 to 1690, into two parts and found that the region containing residues 967 to 1350 is required for its binding to ASF1A (Fig. 3*C*).

We separated ASF1A into two parts (Fig. 3*D*) and performed coimmunoprecipitation experiments with overexpressed SFB-tagged ASF1A FL and mutants in 293T cells. We found that the N-terminal part (residues 1–154), which is the domain that exhibits histone chaperone activity, is required for its interaction with RIF1 (Fig. 3*E*). We then checked whether the histone chaperone activity is required for the interaction with RIF1. We overexpressed the SFB-tagged histone-binding defective mutant (V94R) of ASF1A (71) in 293T cells and carried out coimmunoprecipitation experiments. We found that ASF1A V94R abolished its interaction with histone H3, but this mutant could still bind to RIF1 (Fig. 3*F*). These results suggest that the histone chaperone activity of ASF1A is not required for its interaction with RIF1. We further designed five deletion mutants within the N-terminal ASF1A histone chaperone domain (Fig. S2*A*) to further narrow down the minimal region required for its binding to RIF1. Surprisingly, through the coimmunoprecipitation experiments, we found that none of these mutants could bind to RIF1, three of these mutants lost their ability to bind to H3, whereas two still could bind weakly to H3. Taken together, these findings show that the residues 967 to 1350 region of RIF1 is required for its binding to ASF1A and that the intact histone chaperone domain of ASF1A, but not its histone chaperone activity, is required for its binding to RIF1.

### ASF1 promotes telomere fusion in TRF2-depleted cells and PARPi sensitivity in BRCA1-deficient cells

**Figure 4:**
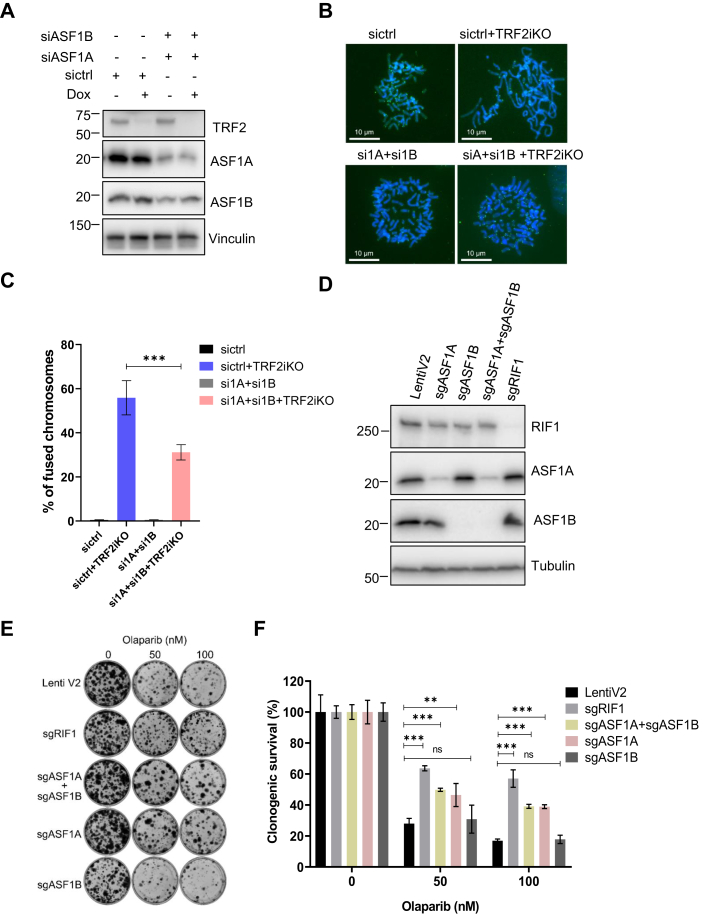
**Loss of ASF1 phenocopies RIF1 deficiency in the regulation of telomere fusion in TRF2-deficient cells and PARPi sensitivity in BRCA1-deficient cells.***A*, Western blot was conducted to determine ASF1A and ASF1B knockdown efficiency as well as TRF2 KO efficiency in inducible TRF2 KO HeLa cells. *B*, representative images of metaphase DNA fluorescence *in situ* hybridization with telomere probe in siControl and siASF1A + siASF1B-transfected inducible TRF2 KO HeLa cells. *C*, statistical quantification of percentage of telomere fusions in inducible TRF2 KO HeLa cells after knockdown of ASF1A and ASF1B (with chromosome counts >1000) from (*A*). Data are represented as mean ± SD (n = 2). ∗∗∗*p* < 0.001 (Student's *t* test). *D*, Western blotting for confirmation of sgASF1A, sgASF1B, and sgRIF1 knockdown efficiency is indicated in sgRNA-transfected stable RPE1 FLAG-Cas9 P53^−/−^ BRCA1^−/−^ cells. *E*, results of long-term clonogenic assays performed using cells from (*D*). About 2 × 10^3^ cells were seeded in triplicate in each well of 6-well plate and incubated with the indicated concentration of olaparib for 14 days. Colonies were fixed and stained with 0.5% crystal violet. *F*, quantification of relative cell survival in the clonogenic assays in (*E*). Data are presented as means (±SD) (n = 3). ∗∗*p* < 0.01; ∗∗∗*p* < 0.001; ns (Student's *t* test). Dox, doxycycline; ns, not significant; PARPi, poly (ADP-ribose) polymerase inhibitor; RIF1, replication timing regulatory factor 1; sgRNA, single-guide RNA; TRF2, telomeric repeat–binding protein 2.

RIF1 acts downstream of 53BP1 to control DSB repair by promoting NHEJ and inhibiting the HR-dependent 5′ end resection (29, 30, 32, 58). RIF1 has been shown to regulate telomere fusion in TRF2-depleted cells and PARPi sensitivity in BRCA1-deficient cells (29, 32, 58). We sought to determine whether ASF1 depletion affects these RIF1-mediated functions. As shown in Figure 4, *A*–*C*, we found that telomere fusion was significantly decreased by knocking down both ASF1A and ASF1B in inducible TRF2 KO HeLa cells compared with control siRNA-treated cells, suggesting a role of ASF1 in the regulation of end joining of dysfunctional telomeres in TRF2-depleted cells. Next, we generated stable LentiV2 mock guide RNA transfected control cells and ASF1A, ASF1B, ASF1A + ASF1B, and RIF1 single-guide RNA (sgRNA) knockdown cells in RPE1 FLAG-Cas9 P53^−/−^ BRCA1^−/−^ cells (Fig. 4*D*) and examined their response to PARPi (olaparib) treatment. As shown in Figure 4, *E* and *F*, similar to RIF1 knockdown, knockdown of ASF1A only or both ASF1A and ASF1B significantly increased the survival of cells treated with PARPi, whereas knockdown ASF1B only did not have the same effect. These data suggest that although ASF1A and ASF1B may have redundant functions, ASF1A plays a dominant role in the response to PARPi in BRCA1-deficient cells.

We further determined whether the binding of ASF1 with RIF1 is required for PARPi resistance in BRCA1-deficient cells. We restored the expression of wildtype ASF1A (with intact H3 and RIF1 binding), ASF1A V94R (with intact RIF1 binding but disrupted H3 binding), ASF1A Δ91 to 120 (with disrupted binding to both H3 and RIF1), or ASF1A 1 to 154 (with intact H3 and RIF1 binding) mutant in RPE1 BRCA1 KO cells with depletion of both ASF1A and ASF1B (Fig. S3*A*). We found that ASF1A Δ91 to 120 could not restore PARPi sensitivity in ASF1A + ASF1B knockdown cells, whereas ASF1A V94R could in a manner similar to that of reconstitution with wildtype ASF1A or ASF1A 1 to 154 mutant (Fig. S3*B*), suggesting that the interaction of ASF1A with RIF1, but not its histone binding activity, is required for its role in regulating PARPi sensitivity in BRCA1-deficient cells.

These data together indicate that ASF1 phenocopies RIF1 in its modulation of telomere fusion in TRF2-deficient cells and sensitivity to PARPi in BRCA1-deficient cells. This activity of ASF1 is dependent on its interaction with RIF1.

### Loss of ASF1 rescues an end resection defect caused by BRCA1 deficiency

**Figure 5:**
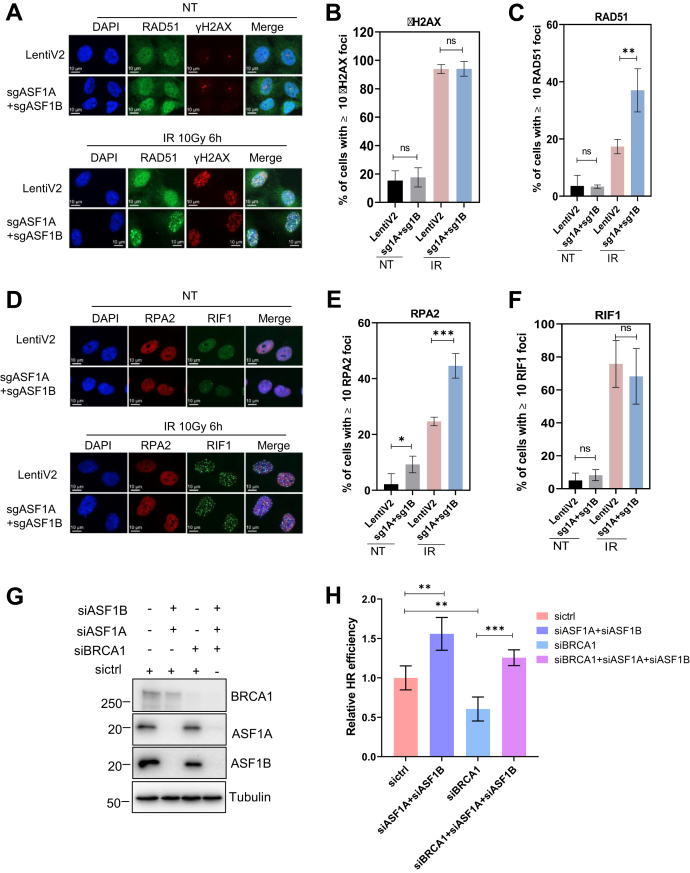
**Loss of ASF1 rescues end resection defect caused by BRCA1 deficiency.***A*, RPE1 FLAG-Cas9 P53^−/−^ BRCA1^−/−^ LentiV2 and sgASF1A + sgASF1B cells were untreated (NT) or treated with 10 Gy of IR and released for 6 h. Cells were fixed and then immunostained with γH2AX and RAD51 antibodies. *B*, statistical quantification of γH2AX foci formation from (*A*). Data are represented as mean (±SD) (n = 3). ns (Student's *t* test). *C*, statistical quantification of RAD51 foci formation from (*A*). Data are represented as mean (±SD) (n = 3). ∗∗*p* < 0.01 (Student's *t* test). *D*, RPE1 FLAG-Cas9 P53^−/−^ BRCA1^−/−^ LentiV2 and sgASF1A + sgASF1B cells were untreated (NT) or treated with 10 Gy of IR and released for 6 h. Cells were fixed and then immunostained with RPA2 and RIF1 antibodies. *E*, statistical quantification of RPA2 foci formation from (*B*). Data are represented as mean ± SD (n = 3), ∗∗∗*p* < 0.001, Student's *t* test. *F*, statistical quantification of RIF1 foci formation from (*B*). Data are represented as mean (±SD) (n = 3). ns (Student's *t* test). *G*, Western blot to determine the siRNA knockdown efficiency in U2OS DR-GFP reporter cell lines transfected with indicated siRNAs. *H*, U2OS DR-GFP reporter cell lines transfected with indicated siRNAs and I-SceI plasmids were collected for flow cytometry analysis to determine HR repair efficiency. Quantification data are represented as mean (±SD) (n = 3). ∗∗*p* < 0.01, ∗∗∗*p* < 0.001 (Student's *t* test). DR, direct repeat; HR, homologous recombination; IR, ionizing radiation; ns, not significant; RPA, replication protein A.

We next examined whether loss of ASF1 in BRCA1-deficient cells could restore HR, which is one of the mechanisms that lead to PARPi resistance. We performed immunostaining to check whether depletion of ASF1 can affect ionizing radiation (IR)-induced RAD51 and RPA2 foci formation, which respectively serve as the markers of HR and end resection, in RPE1 BRCA1 KO cells. We observed that, compared with control cells, depletion of both ASF1A and ASF1B significantly increased the percentage of cells with RAD51 foci upon IR treatment, whereas the percentage of cells with γH2AX foci remained the same (Fig. 5, *A*–*C*), suggesting that ASF1 loss reactivates HR in BRCA1-null cells. Loss of both ASF1A and ASF1B also led to increased IR-induced RPA2 foci formation (Fig. 5, *D* and *E*), suggesting that ASF1 protects DNA ends. We also noticed that depletion of ASF1 did not affect RIF1 foci formation, indicating that ASF1 may act downstream of or in parallel to RIF1 (Fig. 5*F*). Furthermore, HR reporter assay showed that HR repair was partially recovered in BRCA1 knockdown cells with depletion of ASF1A and ASF1B (Fig. 5, *G* and *H*). Taken together, our results indicate that the mechanism of resistance to PARPi in BRCA1-deficient cells upon ASF1 depletion is due to increased end resection and the restoration of HR.

## Discussion

In this study, we identified ASF1 as a player in the 53BP1-dependent pathway. We showed that ASF1 acts with RIF1 to promote telomere fusion in TRF2-depleted cells and suppress HR in BRCA1-deficient cells. The question of how cells strategically and precisely employ the NHEJ and HR DSB repair pathways to help maintain genomic stability has attracted much attention over the past 2 decades. The key pathway involved in this repair pathway choice is the 53BP1-dependent pathway. We now know that 53BP1 acts as an adaptor protein to control two downstream subpathways, which are mediated by RIF1–Shieldin–REV7 and PTIP–Artemis to promote end joining and suppress BRCA1-dependent HR repair, respectively (29, 30, 32, 35, 36, 37, 38, 39, 41, 58). Here, we uncovered a previously unknown component, ASF1, in this process.

In yeasts and metazoans, RIF1 plays critical roles in at least two cellular processes: DNA replication timing and DNA repair (46, 48, 49, 50, 51, 52). However, only a few factors have been identified that act with RIF1 to regulate these functions. To analyze RIF1-associated protein complexes at the endogenous level, we inserted an SFB tag at the N terminus of human RIF1 genomic loci and performed TAP purification of endogenous RIF1. We identified ASF1 as a novel binding partner of RIF1. As mentioned previously, the major function of ASF1 is to act as an upstream H3–H4 histone chaperone that helps the handover of H3.1–H4 histones to histone chaperone CAF-1 for replication-dependent chromatin assembly and the handover of H3.3–H4 histones to histone chaperone HIRA for replication-independent chromatin assembly (59, 60, 61, 62, 63). Several reports also found that ASF1 may participate in the regulation of DNA repair. ASF1A promotes NHEJ repair by facilitating MDC1 phosphorylation by ATM at DSBs (68). ASF1 may also regulate the activation of ATM and DNA-PKcs in response to DSBs (69). ASF1 and CAF-1 help recruit MMS22L/TONSL to ssDNA to load RAD51 during HR in human cells (70). Our findings support these early studies and suggest an additional mechanism of ASF1 in these repair pathways *via* its binding to RIF1.

In this study, we discovered that a previously undefined region, residues 967 to 1350, of RIF1 is required for its interaction with ASF1A. Moreover, the N-terminal chaperone domain of ASF1A, but not its histone chaperone activity, is required for its interaction with RIF1. We also suggested that the binding of ASF1 to RIF1, but not its histone chaperone activity, is critical for its role in regulating PARPi sensitivity in BRCA1-deficient cells. These results are similar to a previous report of the role of ASF1A in DSB repair *via* facilitating MDC1 phosphorylation, which does not require its histone chaperone activity (68). Nevertheless, the whole chaperone domain of ASF1 is required for its binding to RIF1. We also identified that RIF1 itself contains a histone H3 binding region (unpublished data), which may help histone deposition. We propose that ASF1, RIF1, and histone H3 may act together, which on one hand promotes chromatin assembly and on the other hand facilitates proper DSB repair in S-phase cells. The connection between DSB repair choice and chromatin assembly by ASF1 and RIF1 needs to be further investigated.

In summary, our data reveal a crucial role of ASF1 in the regulation of DSB repair choice, which is dependent on its interaction with RIF1. Our discovery of the ASF1–RIF1 association has important implications for the treatment of BRCA1-mutated cancers, as alterations in the ASF1 gene may cause clinical resistance to PARPis.

## Experimental procedures

### Cell lines

293T cells were purchased from the American Type Culture Collection and cultured in Dulbecco’s modified Eagle's medium (DMEM) supplemented with 10% fetal bovine serum. RPE1-hTERT FLAG-Cas9 TP53^−/−^ BRCA1^−/−^ cells were a gift kindly provided by Dr Daniel Durocher (University of Toronto) and were cultured in DMEM with 10% fetal bovine serum. Inducible TRF2 KO HeLa cells were cultured in DMEM with 10% fetal bovine serum.

### Plasmids

DNA fragments corresponding to sgRNAs were cloned into pX330 (Addgene: 42230) for the knock-in of N-terminal SFB tag at endogenous RIF1 locus. LentiCRISPRv2 (Addgene: 52961) was used for ASF1A, ASF1B, and RIF1 knockdown. Donor vector for RIF1 knock-in was generated by Gibson assembly of 5′ homolog arm, Puro-P2A-SFB, and 3′ homolog arm into PUC19 vector. The human RIF1 and ASF1A/ASF1B open reading frames were generated by PCR amplification from complementary DNA prepared from 293T cells. RIF1-truncation mutants, FL ASF1A, FL ASF1B, and the indicated RIF1 or ASF1A domain deletion or point-mutation mutants were subcloned into the pBabe-SFB or modified pLEX_307 SFB vector by Gateway recombination cloning technology.

### Antibodies, siRNAs, and sgRNAs

Antibodies used in this study are 53BP1 (catalog no.: NB100-304; Novus Biologicals), RIF1 (catalog no.: 95558S; Cell Signaling Technology), BRCA1 (catalog no.: sc-6954; Santa Cruz Biotechnology), ASF1A (catalog no.: 2990S; Cell Signaling Technology), ASF1B (catalog no.: 2902S; Cell Signaling Technology), Vinculin (catalog no.: V9131; MilliporeSigma), FLAG (catalog no.: F3165; MilliporeSigma), β-tubulin (catalog no.: T5168; MilliporeSigma), RAD51 (catalog no.: ab63801; Abcam), γH2AX (catalog no.: 05-636l; MilliporeSigma), and RPA2 (catalog no.: 2208S; Cell Signaling Technology).

siRNAs used in this study are control siRNA (catalog no.: 1022076; Qiagen), BRCA1 siRNA (catalog no.: SI02664361; Qiagen), ASF1A siRNA (catalog no.: SI04270182; Qiagen), ASF1B siRNA (catalog no.: SI04278414; Qiagen), ON-TARGETplus Human ASF1A siRNA (catalog no.: L-020222-02-0005; Dharmacon), and ON-TARGETplus Human ASF1B siRNA (catalog no.: L-020553-00-0005; Dharmacon). sgRNAs used in this study for knockdown experiments were ligated to LentiCRISPR V2 (catalog no.: 52961; Addgene) vector. sgRNA sequences are RIF1 sgRNA: GCAGACATTTCCCTCTGAAG; ASF1A sgRNA: CTAATTACTTGTACCTATCG; and ASF1B sgRNA: CTCCTGTCCATGGTAGGTGC.

### SFB tagging of endogenous RIF1

CRISPR–Cas9 technology was used to knock in SFB tag to the N terminus of endogenous RIF1 as previously reported (72). 293T cells were cotransfected with RIF1 knock-in sgRNA and a donor vector containing puromycin resistance selection gene, P2A self-cleavage site, and SFB sequence, flanked by approximately 1 kb of homology arms in PUC19 backbone. After selected with puromycin (2 μg/ml) for 4 days, the remained colonies were seeded in 96-well plate, positive clones were screened by genomic PCR, and further validated by Western blotting and immunofluorescence staining. The RIF1 NKI sgRNA sequence is CCTCAGGGTGGCCGACATGA.

### TAP

293T RIF1 SFB knock-in cells and 293T cells with overexpression of N-terminal SFB-tagged ASF1A or ASF1B were collected for TAP. For the analysis of endogenous RIF1-associated proteins, cells were first lysed in NETN (250 mM NaCl, 1 mM EDTA, 20 mM Tris–HCl [pH 8.0], 0.5% NP-40) buffer and separated into soluble and chromatin fraction as previously described (72). For the analysis of ASF1A- and ASF1B-associated proteins, cells were lysed in NETN buffer with turbonuclease for 1 h and centrifuged at 13,000 rpm for 15 min at 4 °C to get the whole cell lysates. The soluble and chromatin fractions of RIF1, the whole cell lysates of ASF1A or ASF1B were first incubated with streptavidin-conjugated beads (Amersham) for 2 h at 4 °C. After being washed with NETN buffer for three times, proteins binding on the beads were eluted with 2 mg/ml biotin (Sigma–Aldrich) for 1 h. The eluted proteins were then incubated with S-protein beads (Novagen) for 2 h at 4 °C. After being washed with NETN for five times, the bound proteins were boiled in 2× Laemmli buffer, resolved by SDS-PAGE and stained with Coomassie brilliant blue.

### Mass spectrometry analysis

The Coomassie brilliant blue–stained gel samples were excised and destained completely. Then in-gel digestion was performed with trypsin (Promega Corporation) in 50 mM NH_4_HCO_3_ at 37 °C. After 24 h, the digested peptides were extracted and vacuum dried. The samples were reconstituted in the mass spectrometry (MS) loading solution (2% acetonitrile and 0.1% formic acid) before MS analysis.

MS sample was separated by nano–reverse-phase high-performance liquid chromatography and eluted with acetonitrile gradient from 5% to 35% for 60 min at a flow rate of 300 nl/min. The elute was analyzed by the Q Exactive HF MS system (Thermo Fisher Scientific) with positive ion mode and data-dependent manner. The full MS scan was performed with a scanning range of *m/z* 350 to 1200 and resolution at 60,000 at *m/z* 400. After one full scan, up to 20 MS/MS scans followed.

The raw MS data were accessed with Proteome Discoverer 2.2 (Thermo Fisher Scientific) to generate peak list and submitted to Mascot 2.5 (Matrix Science) for database search. The database was *Homo sapiens* downloaded from UniProt (July 2020) with 20,383 entries in total. Tolerance of two missed trypsin cleavages was applied. Variable modifications included carboxyamidomethyl for cysteine and oxidation for methionine. The mass tolerance settings are 10 ppm for precursor ion and 0.02 Da for product ion. Percolator algorithm was applied to examine the MS analysis and false discovery rate lower than 1%.

### Coimmunoprecipitation or pull-down assays

For all coimmunoprecipitation and pull-down assays conducted in this study, cells were lysed in NETN buffer, with phosphatase and proteinase inhibitors, and 50 U turbonuclease) for 1 h and centrifuged at 13,000 rpm for 15 min at 4 °C. For pull down with endogenous antibodies, the supernatant was collected and incubated with the indicated antibodies overnight and then incubated with Protein A/G agarose beads (Santa Cruz Biotechnology) for 2 h at 4 °C; for pull down with SFB-tagged proteins, the supernatant was incubated with streptavidin-conjugated beads (Amersham) or S-protein beads (Novagen) for 2 h at 4 °C. After being washed with NETN for three times, the bound proteins were boiled in 2× Laemmli buffer, resolved by SDS-PAGE, and blotted with the indicated antibodies.

### Clonogenic survival assay

About 2 × 10^3^ cells each for indicated Lenti-sgRNA transfected RPE1-hTERT TP53^−/−^ BRCA1^−/−^ cells used in the study were seeded onto 6-well plates in triplicate, treated with various doses (0, 50, and 100 nM) of Olaparib (BioVision), and then incubated for 14 days. Colonies were fixed and stained with 0.5% crystal violet. Relative cell viability was measured using ImageJ (National Institutes of Health).

### CellTiter-Glo assay

Five hundred cells in a volume of 100 μl were plated into each well of 96-well plates on day 0. After 24 h, 100 μl of the 2× dilution of olaparib was added to the cells in technical triplicates. After 7 days of incubation, cell viability was measured using a CellTiter-Glo luminescent cell viability assay (catalog no.: G7572; Promega) with a BioTek Synergy 2 Multimode Microplate Reader.

### Immunofluorescence staining

Immunostaining assays were performed as described previously (30). Briefly, cells seeded on glass coverslips were first fixed in 4% formaldehyde for 10 min. After washed with PBS for twice, cells were then permeabilized in 0.5% Triton X-100 for 15 min. After blocking with 5% bovine serum albumin for 1 h, cells were then incubated with indicated primary antibodies for 2 h at 37 °C and secondary antibodies for 1 h at room temperature. After being washed with PBS for three times, coverslips were dried and mounted with 4′,6-diamidino-2-phenylindole solution (Thermo Fisher Scientific).

### Telomere fusion analysis

TRF2-inducible KO HeLa cells transfected with indicated siRNAs were first treated with or without 0.5 μg/ml doxycycline for 6 days to induce TRF2 depletion. Detailed procedures were done as described previously (72). For each group, a minimum of 1000 chromosomes were counted for analysis.

### HR reporter assays

U2OS cells stably expressing HR reporter (direct repeat–GFP reporter) were used to determine DSB repair efficiency by HR as previously described (73). Briefly, U2OS direct repeat-GFP cells were transfected with same amount of control siRNA, ASF1A + ASF1B siRNAs, BRCA1 siRNA, or ASF1A + ASF1B siRNAs + BRCA1 siRNA with Lipofectamine RNAiMAX (Thermo Fisher Scientific). About 24 h later, 2 μg of pCBASce plasmid (I-SceI expression vector) were transfected into each cells with Lipofectamine 3000 (Thermo Fisher Scientific). After culture for another 48 h, cells were subjected to flow cytometry analysis to determine the percentage of GFP-positive cells.

### Statistical analyses

Statistical analyses were performed by using two-tailed Student's *t* tests or one-way ANOVA in GraphPad Prism 8 (GraphPad Software, Inc). All results are presented as means ± SD, and all experiments were repeated at least two times. *p* Value <0.05 was considered statistically significant.

## Data availability

The MS proteomics data have been deposited to the ProteomeXchange Consortium (http://www.ebi.ac.uk/pride) with the dataset identifier PXD027727.

## Supporting information

This article contains supporting information.

## Conflict of interest

The authors declare that they have no conflicts of interest with the contents of this article.
