# Supplementary figures and images for: Histone chaperone ASF1 acts with RIF1 to promote DNA end joining in BRCA1-deficient cells
Source: J Biol Chem. 2022 Apr 25;298(6):101979. doi: 10.1016/j.jbc.2022.101979 (PMC9127577; doi:10.1016/j.jbc.2022.101979)

**A**

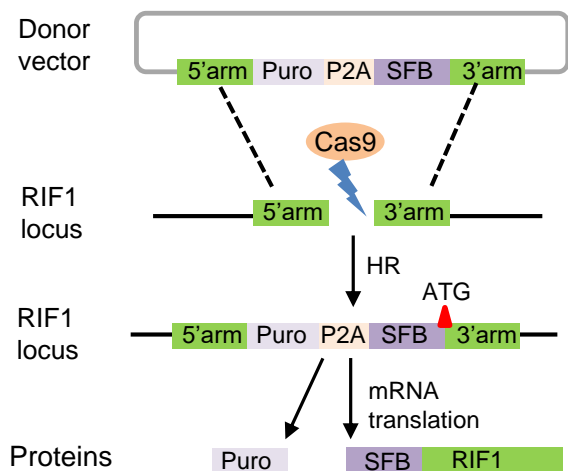

**B**

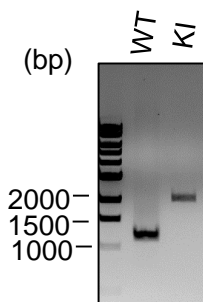

**C**

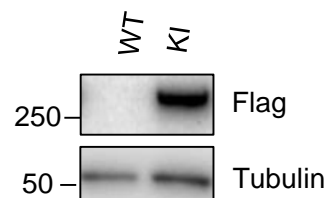

**D**

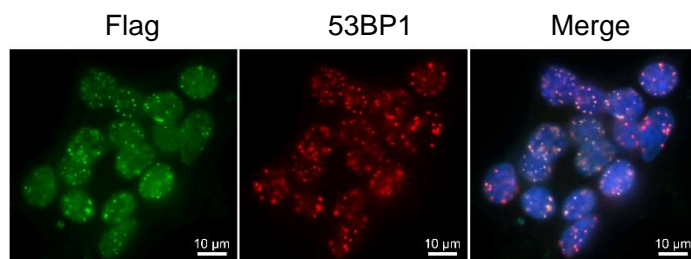

A

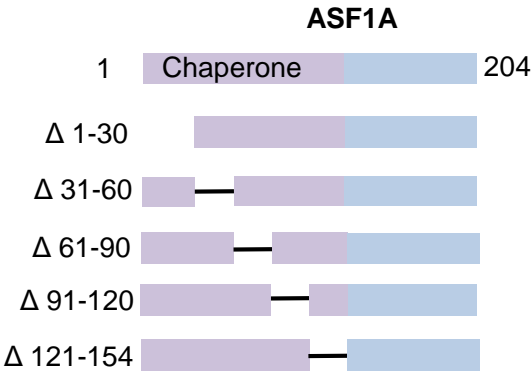

B

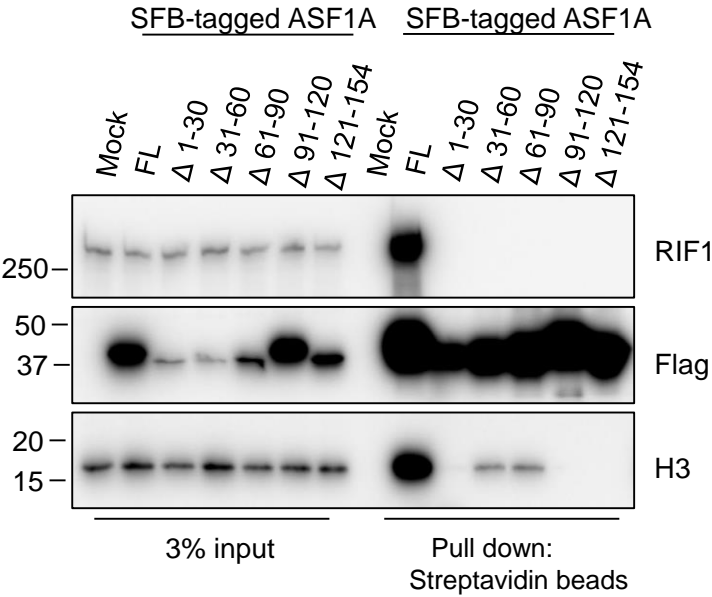

Figure S3

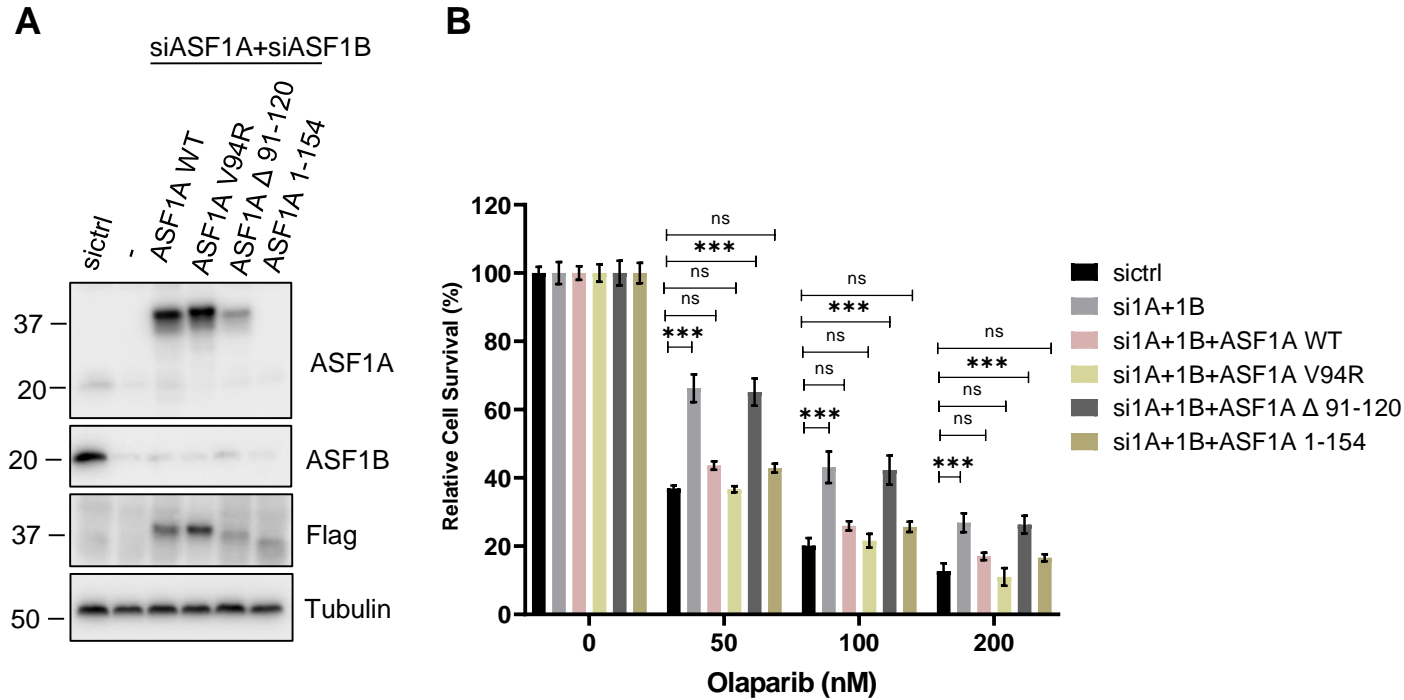

Supplement: Supplementary Figures S1–S3 [file mmc4.pdf]
